# Comparison of the impact of exercise training combined with dietary intervention vs. dietary intervention alone in patients with obesity and metabolic syndrome—a systematic review

**DOI:** 10.3389/fnut.2025.1703600

**Published:** 2025-12-08

**Authors:** Youxiang Cao, Rui Xu, Jie Zhang

**Affiliations:** 1 School of Physical Education, Nanjing Xiaozhuang University, Nanjing, China; 2 School of Sports and Health, Nanjing Sport Institute, Nanjing, China; 3 Laboratory of Kinesiology, Nanjing Sport Institute, Nanjing, China

**Keywords:** diet intervention, exercise training, metabolic syndrome, obesity, weight loss

## Abstract

**Background:**

Metabolic syndrome (MetS) is characterized by a cluster of metabolic risk factors, including abdominal obesity, raised triglycerides (TGs), lowered high-density lipoprotein cholesterol (HDL-c), hypertension, and impaired glucose tolerance. Multifaceted interventions, including dietary intervention (DI) and exercise training (Ex), are recommended for the treatment of MetS.

**Objective:**

Our study is a systematic review and meta-analysis comparing the efficacy of DI alone vs. DI with Ex (DI + Ex) on body composition, blood pressure (BP), and other cardiometabolic risk factors in patients with obesity and MetS.

**Method:**

We searched PubMed, Web of Science, SPORTDiscus, and the Cochrane Library from their inception to 10 August 2025, for randomized controlled trials. We calculated mean differences (MD) or standardized mean differences (SMD) and 95% confidence intervals (CIs) using random- or fixed-effect models (depending on heterogeneity). The major outcome was improvement in MetS risk factors, including changes in waist circumference (WC), TGs, HDL-c, BP, and fasting plasma glucose (FPG). The secondary outcomes were body weight, body mass index (BMI), body fat (BF), fasting insulin, total cholesterol (TC), low-density lipoprotein cholesterol (LDL-c), and glycated hemoglobin (HbA1c).

**Results:**

In this meta-analysis of 16 studies involving 902 individuals with obesity and MetS, the DI + Ex group demonstrated a significant advantage over DI for improving specific MetS risk factors. Specifically, the DI + Ex group showed superior efficacy in reducing WC [MD = 2.11 cm, 95% CI: (0.99, 3.23)] and FPG [SMD = 0.22, 95% CI: (0.03, 0.40)]. However, the addition of exercise did not confer a significant benefit for HDL-c, BP, or TG. Beyond the primary MetS factors, the combined intervention was also more effective for a range of secondary outcomes, including body weight, BMI, BF, TC, and LDL-c.

**Conclusion:**

Our findings demonstrate that DI + Ex interventions yield significant improvements in multiple MetS risk factors among individuals with obesity. We recommend that healthcare providers and public health initiatives prioritize these integrated programs to optimize cardiometabolic health outcomes in this population.

## Introduction

Metabolic syndrome (MetS) is a major risk factor for cardiovascular diseases, type 2 diabetes, and other negative health outcomes, posing a major challenge to clinical practice and public health (1). Against the backdrop of current lifestyles and dietary habits, the incidence of MetS is increasing annually, a trend that is particularly pronounced among obese populations (2, 3). Although the current understanding of the pathogenesis of MetS remains incomplete, research suggests that it results from the interaction of factors, such as obesity, high-calorie diets, and lack of physical exercise (4, 5). The main characteristics of MetS include obesity, insulin resistance, hypertension, hyperglycemia, and dyslipidemia; these characteristics also form the defining criteria for this condition (5, 6).

The core of MetS is obesity-related glucose and lipid metabolic disorders. Therefore, current non-surgical intervention strategies primarily focus on achieving a negative energy balance, mainly through exercise training (Ex) and dietary intervention (DI). Achieving a negative energy balance through Ex or increased physical activity levels is an important clinical approach for obesity management or health maintenance (7). In recent years, DI has gained increasing attention. Caloric restriction through DI can effectively improve MetS by enhancing insulin sensitivity and regulating lipid metabolism, among other benefits (8–10). Studies indicate that dietary caloric restriction appears to be more effective for weight management than Ex (7) but may lead to a reduction in lean body mass and basal metabolic rate (11). By contrast, Ex is effective in preserving lean body mass during weight loss (12). For obese individuals—particularly those who belong to exercise-based weight-loss plans—adhering to the weight-loss programs is often difficult (13). Consequently, an increasing number of studies are exploring the combination of DI and Ex (DI + Ex) to mitigate the drawbacks of single interventions and achieve synergistic benefits. Although several meta-analyses have investigated the effects of DI alone vs. those of DI + Ex on blood lipids (14), inflammatory markers (15), blood glucose (16), and body composition (17) in populations with obesity and MetS, no studies have compared DI alone with DI + Ex specifically in individuals with obesity and MetS.

In this study, we explored the intervention effects of DI alone vs. those of DI + Ex in individuals with obesity and MetS.

## Methods

This review was performed in accordance with the Preferred Reporting Items for Systematic Reviews and Meta-analysis (PRISMA) (18).

### Types of studies

Randomized controlled trials (RCTs) were eligible.

### Control

All studies compared DI with Ex+DI, and their outcomes were measured at pre- and post-intervention.

### Literature search

Several databases, including PubMed, Web of Science, SPORTDiscus, and the Cochrane Library, were searched to identify relevant RCTs published from database inception to 10 August 2025, by two investigators (Cao and Xu). The search strategy involved a Boolean search using a combination of subject-related and free words to identify pertinent data in titles, abstracts, and keywords. The following search terms were used: (“exercise” OR “physical activity” OR “aerobic training” OR “AT” OR “combine training” OR “CT” OR “resistance training” OR “RT” OR “high-intensity interval training” OR “HIIT”) AND (“dietary intervention” OR “caloric restriction” OR “lifestyle intervention) AND (“obese” OR “obesity”) (Supplementary File).

### Study selection

The selection criteria for this meta-analysis were as follows: (a) The participants were adults (age > 18 years) with MetS (incorporating the definitions of the International Diabetes Federation and the American Heart Association/National Heart, Lung, and Blood Institute) (19). (b) The intervention program was conducted between DI and DI + Ex and lasted for at least 2 weeks. (c) The studies were RCTs or had parallel study designs. (d) The reported outcomes included waist circumference (WC), triglycerides (TGs), high-density lipoprotein cholesterol (HDL-c), blood pressure (BP), fasting plasma glucose (FPG), and fasting insulin (each study included at least three of these outcomes), with measurements taken at pre- and post-intervention. (e) Articles were in the English language. Exclusion criteria were as follows: (a) Non-original research. (b) Studies with unreliable designs or substantial statistical errors. (c) Interventions with Ex, DI, or Ex+DI only or other interventions. (d) Participants with cancer, HIV, chronic heart and/or liver failure, or other conditions limiting their ability to perform Ex or DI. (e) Articles in non-English languages. In the case of DI, studies included used any type of DI. In the case of Ex, studies involving any type of Ex or physical activity were included.

### Measured outcomes

The major outcomes were the improvement in MetS, including changes in WC, TG, HDL-c, BP, and FPG levels. Secondary outcomes were body weight (BW), body mass index (BMI), body fat (BF), fasting insulin (FINs), total cholesterol (TC), low-density lipoprotein cholesterol (LDL-c), and glycated hemoglobin (HbA1c).

### Data extraction

Two researchers (Cao and Xu) extracted data and research characteristics from the qualifying literature, and disagreements were resolved by discussion with another reviewer. Duplicate studies were excluded from different databases. The extracted content from the literature included the following: first author, publication year, country, participant characteristics (sample size, age, and sex), intervention characteristics, and outcomes. Raw pre- and post-intervention values or differences (post-intervention values − pre-intervention values) were extracted for main variable measurements. Values presented as quartiles, confidence intervals, median, or standard errors were all converted into means and standard deviations (20–22).

### Assessment of study quality

Study quality was independently assessed by two investigators (Cao and Zhang) using the revised Cochrane risk-of-bias tool for randomized trials (RoB 2) (23). The RoB 2 tool was employed to assess the following variables: (I) bias arising from randomization, (II) bias because of deviations from intended interventions, (III) bias because of missing data, (IV) bias in outcome measurement, and (V) bias in the selection of the reported outcomes. Any discrepancy was resolved by consensus. The Grading of Recommendations Assessment, Development, and Evaluation (GRADE) system was applied to assess the quality of the evidence (24).

### Heterogeneity

Heterogeneity between trials was analyzed through the *χ*^2^ test, and the test level of *p*<0.1 was regarded as indicating significant heterogeneity. Cochran Q statistics were calculated, and the following formula was utilized to calculate I^2^, which was used to qualify the level of heterogeneity (25). The magnitude of heterogeneity (I^2^) was categorized as low (0–24%), moderate (25–49%), substantial (50–74%), and considerable (75–100%) (25).


$$I^{2}=\frac{Q-\mathrm{df}}{Q}$$

### Data analysis

Meta-analyses were conducted by employing Review Manager software (version 5.3; The Nordic Cochrane Center, London, United Kingdom) or Statistics and Data Science software (version 17.0; Stata Corporation). Measurement data were calculated as mean differences (MD) or standardized mean differences (SMD) by using random or fixed-effects models (fixed models were used when I^2^ was less than 25%, whereas random models were applied when *I*^2^ was higher than 25%). In addition, for SMDs, effect size analysis statistics were considered as small (0.2), medium (0.5), and large (0.8) (26). The precision of the pooled effect size was reported as 95% confidence intervals (CI). Sources of heterogeneity were identified through subgroup analyses based on exercise type, duration, and year. If statistical heterogeneity existed among the results, the source of heterogeneity was further analyzed by using sensitivity analyses, which were conducted by removing each study individually. All analyses were conducted separately for each outcome variable.

## Results

### Literature search

**Figure 1:**
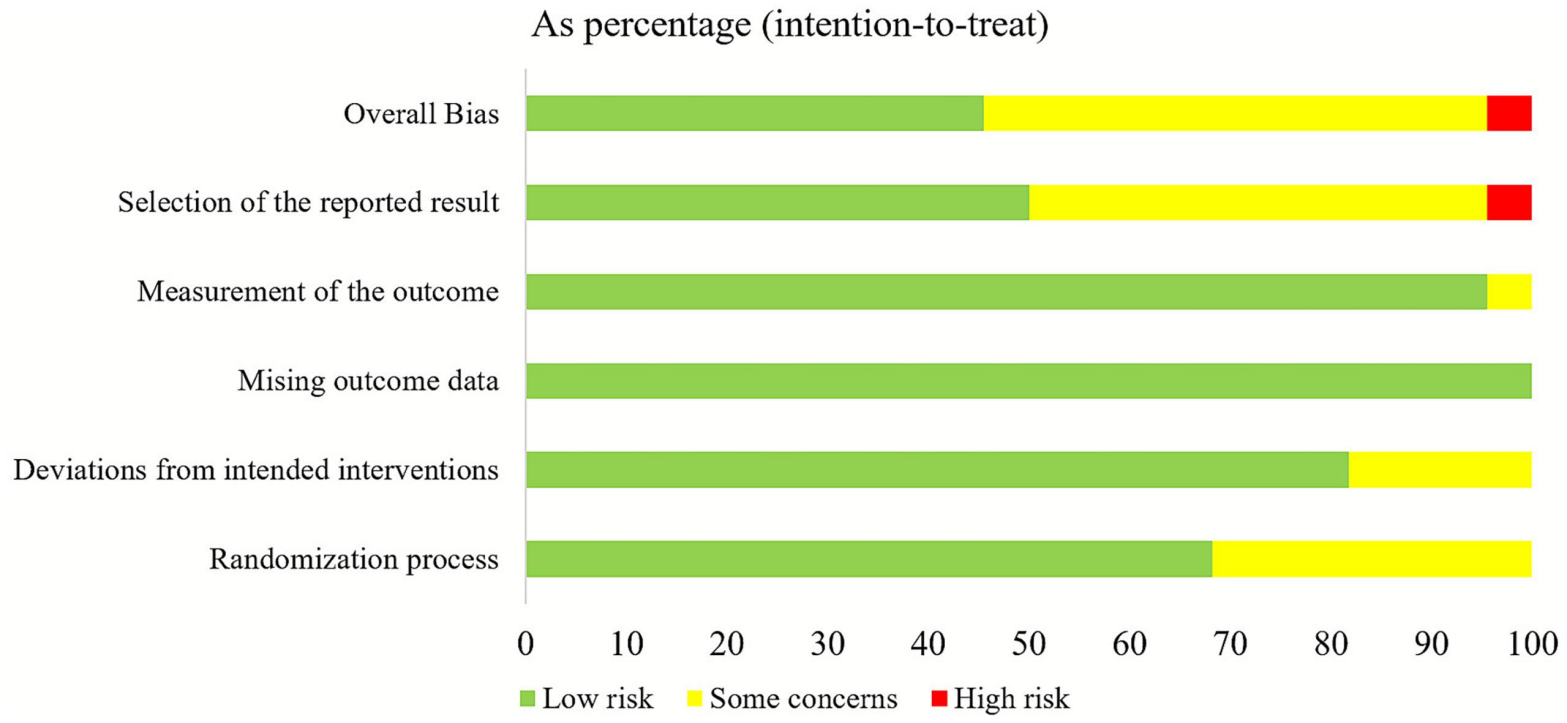
PRISMA flow diagram.

We identified a total of 18,493 potential studies across PubMed, Cochrane Library, Web of Science, and SPORTDict. After removing duplicates and eliminating papers on the basis of the eligibility criteria, 190 articles for full-text checking remained. After reviewing the full texts, we excluded 174 studies that did not meet the requirements. Finally, 16 studies met the selection criteria (27–42). The details of the search are shown in Figure 1.

### Included trials

We included 16 studies (22 trials) with 840 participants in our meta-analysis. All of these participants were diagnosed as obese with MetS. The largest number of participants in single studies was 90 (31), and the smallest was 22 (28). Three studies included only male participants (29, 36), and three studies included only female participants (27, 28). Two studies were conducted with resistance training (RT) + DI (27, 29). Four studies involved high-intensity interval training (HIIT) + DI (39, 41, 42); four studies involved combined training (CT) + DI (34, 36, 38), and 14 studies included aerobics training (AT) + DI (27–33, 35, 37, 40, 41). The mean age of the participants ranged from 19 to 70 years, and the publication dates of the articles ranged primarily from 2002 to 2025. The detailed characteristics of participants are summarized in Table 1.

**Table 1:** Characteristics of the participants and exercise intervention.

Author, year	Country	MetS condition	Sample size [total, male (%)]	Age [years]	Study duration (weeks)	DI detail	Exercise detail
Bagnato et al. (41)	Italy	①③⑤	DI: 17, 9(53%) DI + Ex:12, (6)50%	DI: 51.2 ± 10.52 DI + Ex: 51.2 ± 10.52	8	Healthy eating guidelines, based on the low-glycemic Mediterranean diet	60–75% HRmax, 60 min, 4 sessions/week, aerobic training
Bagnato et al. (41)	Italy	①③⑤	DI:17, 9(53%) DI + Ex: 13, 4(31%)	DI: 51.2 ± 10.52 DI + Ex: 51.2 ± 10.52	8	Healthy eating guidelines, based on the low-glycemic Mediterranean diet	>85% HRmax, 50 min, 3 sessions/week, HIIT
Bagnato et al. (41)	Italy	①③⑤	DI: 17, 9(53%) DI + Ex: 12, 6(50%)	DI: 51.2 ± 10.52 DI + Ex: 51.2 ± 10.52	16	Healthy eating guidelines, based on the low-glycemic Mediterranean diet	60-–75% HRmax, 60 min, 4 sessions/week, aerobic training
Bagnato et al. (41)	Italy	①③⑤	DI: 17, 9(53%) DI + Ex: 13, 4(31%)	DI: 51.2 ± 10.52 DI + Ex: 51.2 ± 10.52	16	Healthy eating guidelines, based on the low-glycemic Mediterranean diet	>85% HRmax, 50 min, 3 sessions/week, HIIT
Battillo et al. (39)	USA	①③④	DI: 12, 0 (0%) DI + E: 11, 0 (0%)	DI: 48.4 ± 2.6 DI + Ex: 47.6 ± 4.3	2	Low-calorie diet, 1,000–1,200 kcal/day	3-min intervals at 50 and 90% HRpeak, total 60 min, HIIT
Bouchonville et al. (34)	USA	Article reported	DI: 26, 9(35%) DI + Ex: 28, 12(43%)	DI: 70 ± 4 DI + Ex: 70 ± 4	26	Caloric restriction of 500–750 kcal/day	90 min, 3 sessions/week, combined training
Bouchonville et al. (34)	USA	Article reported	DI: 26, 9(35%) DI + Ex: 28, 12(43%)	DI: 70 ± 4 DI + Ex: 70 ± 4	52	Caloric restriction of 500–750 kcal/day	90 min, 3 sessions/week, combined training
Brennan et al. (37)	USA	①④⑤	DI: 31, 8(26%) DI + Ex: 28, 8(29%)	DI: 69.1 ± 5.2 DI + Ex: 66.9 ± 3.9	26	Caloric restriction of 500–1,000 kcal/day	50–80% HRR, 45 min, 4–5 sessions/week, aerobic training
Cooper et al. (31)	USA	①③④	DI: 45, 2(4%) DI + Ex: 45, 8(18%)	DI: 47.5 ± 6.2 DI + Ex: 46.8 ± 6.5	52	Low caloric diet, 1,200–2,100 kcal/day	60 min, 5 sessions/week, aerobic training
Ezpeleta et al. (40)	USA	①④⑤	DI: 20, 4(20%) DI + Ex: 20, 3(15%)	DI: 44 ± 16 DI + Ex: 44 ± 13	13	600 kcal/2500 kJ “fast day” alternated with an ad libitum intake “feast day”	65–80% HRmax, 5 sessions/week, 60 min, aerobic training
Fernández et al. (32)	Spain	Article reported	DI: 20, 6(30%) DI + Ex: 20, 7(35%)	DI: 57.2 ± 4.29 DI + Ex: 59.05 ± 5.47	12	Hypocaloric Mediterranean diet (restriction of 500 kcal/day)	65–80% HRmax, 3 sessions/week, 30–60 min, aerobic training
Fernández-García et al. (35)	Spain	①②③④⑤	DI: 37, 19(51%) DI + Ex: 38, 19(50%)	DI: 65.6 ± 4.1 DI + Ex: 63.3 ± 4.4	26	Hypocaloric Mediterranean diet (restriction of 600 kcal/day)	Aerobic training
Folope et al. (38)	France	Article reported	DI: 20, 8(40%) DI + Ex: 20,5(25%)	DI: 39 (32; 44)* DI + Ex: 44(40; 52)*	26	21 g of leucine and 9 g of arginine per day	40—80 min, 3 sessions/week, combined training
Giannopoulou et al. (28)	USA	①②⑤	DI: 11, 0(0%) DI + Ex: 11, 0(0%)	DI: 58.5 ± 1.7 DI + Ex: 57.4 ± 1.7	14	High-monounsaturated fat diet (40% fat, 40% carbohydrates, 20% protein), 2,510 kJ/day	65–70% VO2peak, 60 min, 3–4 sessions/week, aerobic training
Janssen et al. (27)	Canada	①②③	DI: 13, 0(0%) DI + Ex: 11, 0(0%)	DI: 40.1 ± 6.7 DI + Ex: 37.5 ± 6	16	Caloric restriction by 1,000 kcal/day	50–85% HRmax, 5 sessions/week, 60 min, aerobic training
Janssen et al. (27)	Canada	①②③	DI: 13, 0(0%) DI + Ex: 14, 0(0%)	DI: 40.1 ± 6.7 DI + Ex: 34.8 ± 5.8	16	Caloric restriction by 1,000 kcal/day	8–12 RM, 3 sessions/week, 30 min, resistance training
Kukkonen-Harjula et al. (29)	Finland	Article reported	DI: 30, 30(100%) DI + Ex: 30, 30(100%)	DI: 42 (34–50)^#^ DI + Ex: 42 (34–50)^#^	39	Low-energy diet, 5 MJ/day	60–70% VO2max, 3 sessions/week, 45 min, aerobic training
Kukkonen-Harjula et al. (29)	Finland	Article reported	DI: 30, 30(100%) DI + Ex: 30, 30(100%)	DI: 42 (34–50)^#^ DI + Ex: 42 (34–50)^#^	39	Low-energy diet, 5 MJ/day	60–80% of 1 RM with 8 repetitions and 3 sets in each exercise, resistance training
Said et al. (36)	Saudi Arabia	Article reported	DI: 9, 9(100%) DI + Ex: 14, 14(100%)	DI: 19–24^#^ DI + Ex: 19–24^#^	12	Caloric restriction of 500 kcal/day	60–75% HRmax, 40 min, 3session/week, combined training
Snel et al. (33)	Netherlands	①②③④⑤	DI: 14, 6(43%) DI + Ex: 13, 8(62%)	DI: 56.1 ± 2.4 DI + Ex: 53 ± 2.5	16	Very low caloric diet (a total of approximately 450 kcal/day)	70% HRmax, at least 4 sessions/week, 30–60 min, aerobic training
Straznicky et al. (30)	Australia	Article reported	DI: 20, 12(60%) DI + Ex: 20, 12(60%)	DI: 55 ± 1 DI + Ex: 55 ± 1	12	Caloric restriction by 1,600 kcal/day	65% HRmax, 40 min, alternate days, aerobic training
Suárez-Cuenca et al. (42)	Mexico	Article reported	DI: 14, 3(21%) DI + Ex: 12, 2(17%)	DI: 56.7 (51–65)* DI + Ex: 54(45–56.7)*	12	Mediterranean diet	3 sessions/week, HIIT

DI, Dietary intervention; Ex, Exercise intervention; HIIT, High-intensity interval training; HRmax, Maximal heart rate; MJ, Megajoule; RM, Repetitive maximum; VO2peak, Peak oxygen consumption. ① WC ② TG ③ HDL-c ④ SBP and/or DBP ⑤ FBP. *Data were expressed as the median (quartiles 1 and 3). #Data were expressed as intervals (minimum value and maximum value).

### Risk of bias among the included trials

**Figure 2:**
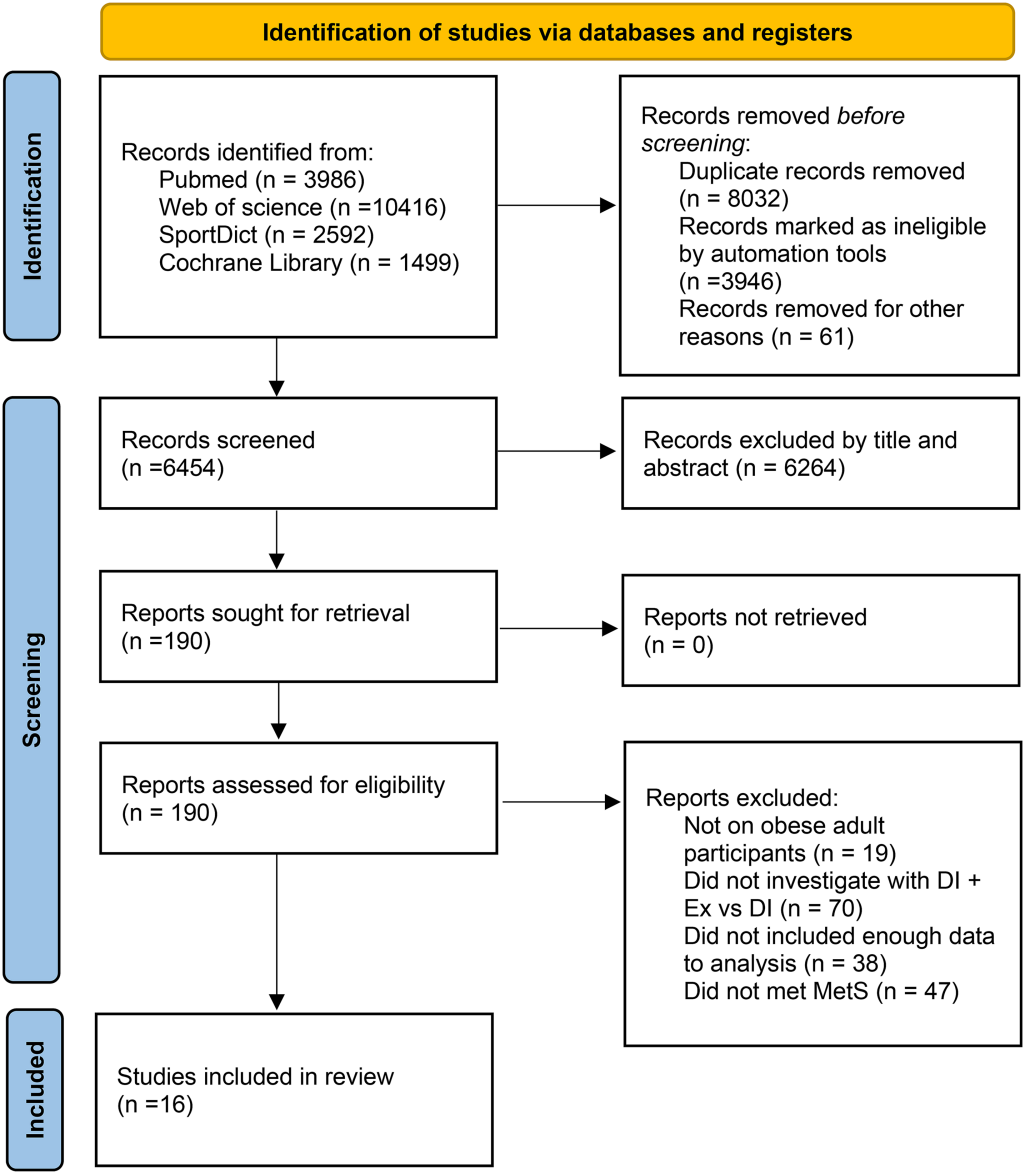
Risk of bias summary for the included studies.

**Figure 3:**
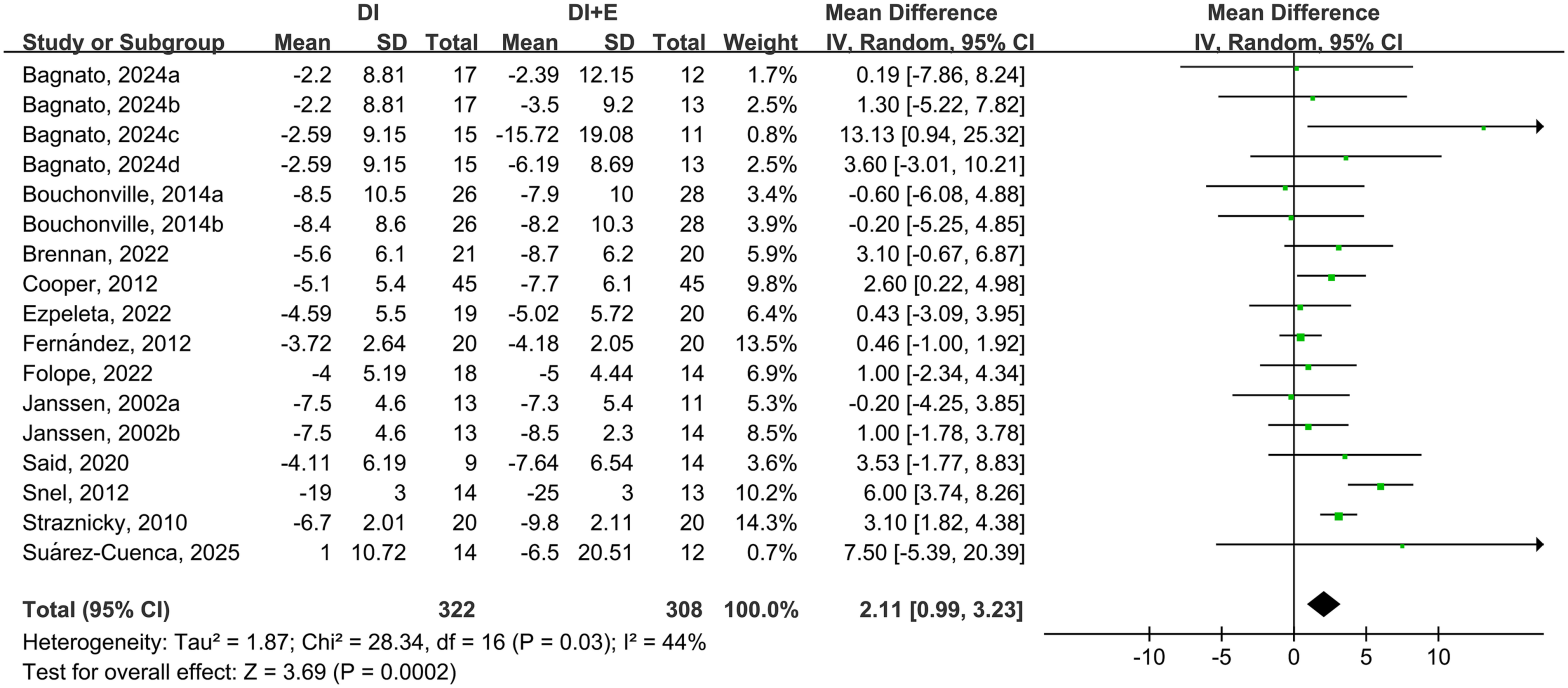
Risk of bias graph.

We assessed 22 trials for risk of bias, and the results are shown in Figures 2, 3. All studies were deemed to be at low risk of bias for the missing outcome data domain, and the selection of the reported results domain was rated as low risk or of some concern. The majority of the studies were considered to be of some concern regarding the risk of bias for the rest of the items.

## Meta-analysis of overall effects

### Effect of DI vs. DI + ex on risk factors for mets

**Figure 4:**
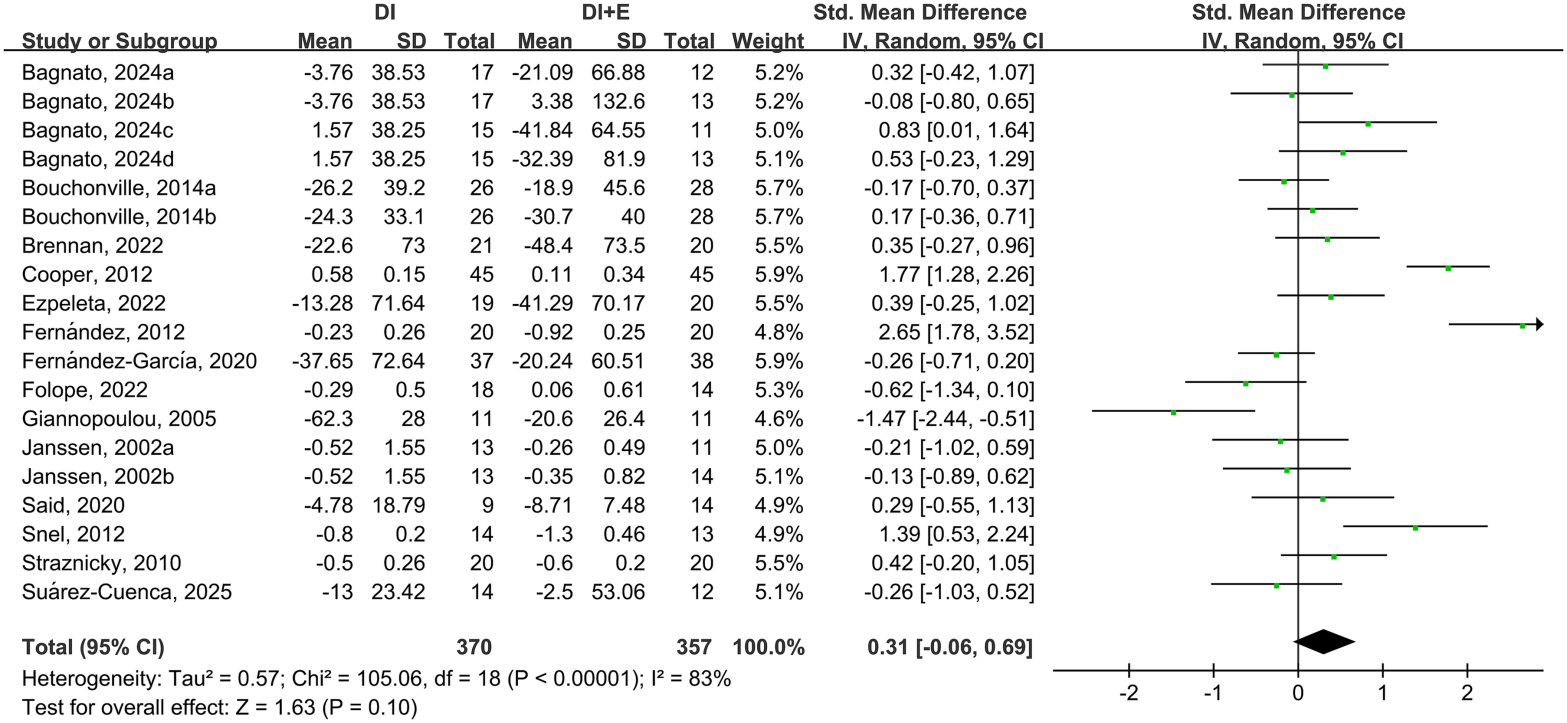
Effect of DI vs. DI + E intervention on WC.

The within-group analysis found that DI [MD = −5.69 cm, 95% CI: (−8.63, −2.75), *p* < 0.001, *I*^2^ = 89%] and DI + Ex [MD = −8.29 cm, 95% CI: (−12.06, −4.51), *p* < 0.001, *I*^2^ = 94%] resulted in significant improvements in WC (Table 2). Compared with DI, DI + Ex showed greater benefits for WC [MD = 2.11 cm, 95% CI: (0.99, 3.23), *p* = 0.03, *I*^2^ = 44%] (Figure 4).

**Table 2:** Effect of DI vs. DI + Ex intervention on MetS risk factors.

Outcomes	Statistical indicators		Within-group		DI vs. DI + Ex
			DI	DI + Ex	
WC (cm)	Trails		17	17	17
	MD (95% CI)		−5.69 [−8.63, −2.75]	−8.29 [−12.06, −4.51]	2.11 [0.99, 3.23]
	Heterogeneity	*I* ^2^	89	94	44
		*p*	<0.001	<0.001	0.03
	*p*		<0.001	<0.001	<0.001
TG	Trails		19	19	19
	MD (95% CI)		−0.46 [−0.85, −0.07]	−0.77 [−1.15, −0.39]	0.31 [−0.66, 0.69]
	Heterogeneity	*I* ^2^	85	82	83
		*p*	<0.001	<0.001	<0.001
	*p*		0.02	<0.001	0.10
FPG	Trails		22	22	22
	MD (95% CI)		−0.55 [−0.91, −0.19]	−0.78 [−1.13, −0.43]	0.22 [0.03, 0.40]
	Heterogeneity	*I* ^2^	84	82	
		*p*	<0.001	<0.001	<0.001
	*p*		<0.001	<0.001	
SBP	Trails		13	13	13
	MD (95% CI)		−7.92 [−12.49, −3.35]	−6.81 [−11.40, −2.23]	−0.68 [−3.27, 1.91]
	Heterogeneity	*I* ^2^	91	91	66
		*p*	<0.001	<0.001	<0.001
	*p*		<0.001	<0.001	0.61
DBP	Trails		13	13	13
	MD (95% CI)		−4.50 [−6.16, −2.84]	−4.84 [−7.41, −2.28]	0.33 [−2.72, 3.38]
	Heterogeneity	*I* ^2^	70	89	92
		*p*	<0.001	<0.001	<0.001
	*p*		<0.001	<0.001	0.83
HDL-c	Trails		21	21	21
	MD (95% CI)		−0.08 [−0.37, 0.21]	0.13 [−0.11, 0.38]	−0.14 [−0.36, 0.08]
	Heterogeneity	*I* ^2^	76	66	57
		*p*	<0.001	<0.001	<0.001
	*p*		0.58	0.29	0.22

DBP, Diastolic blood pressure; FPG, Fasting plasma glucose; HDL-c, High-density lipoprotein cholesterol; SBP, Systolic blood pressure; WC, Waist circumference; TG, Triglycerides.

**Figure 5:**
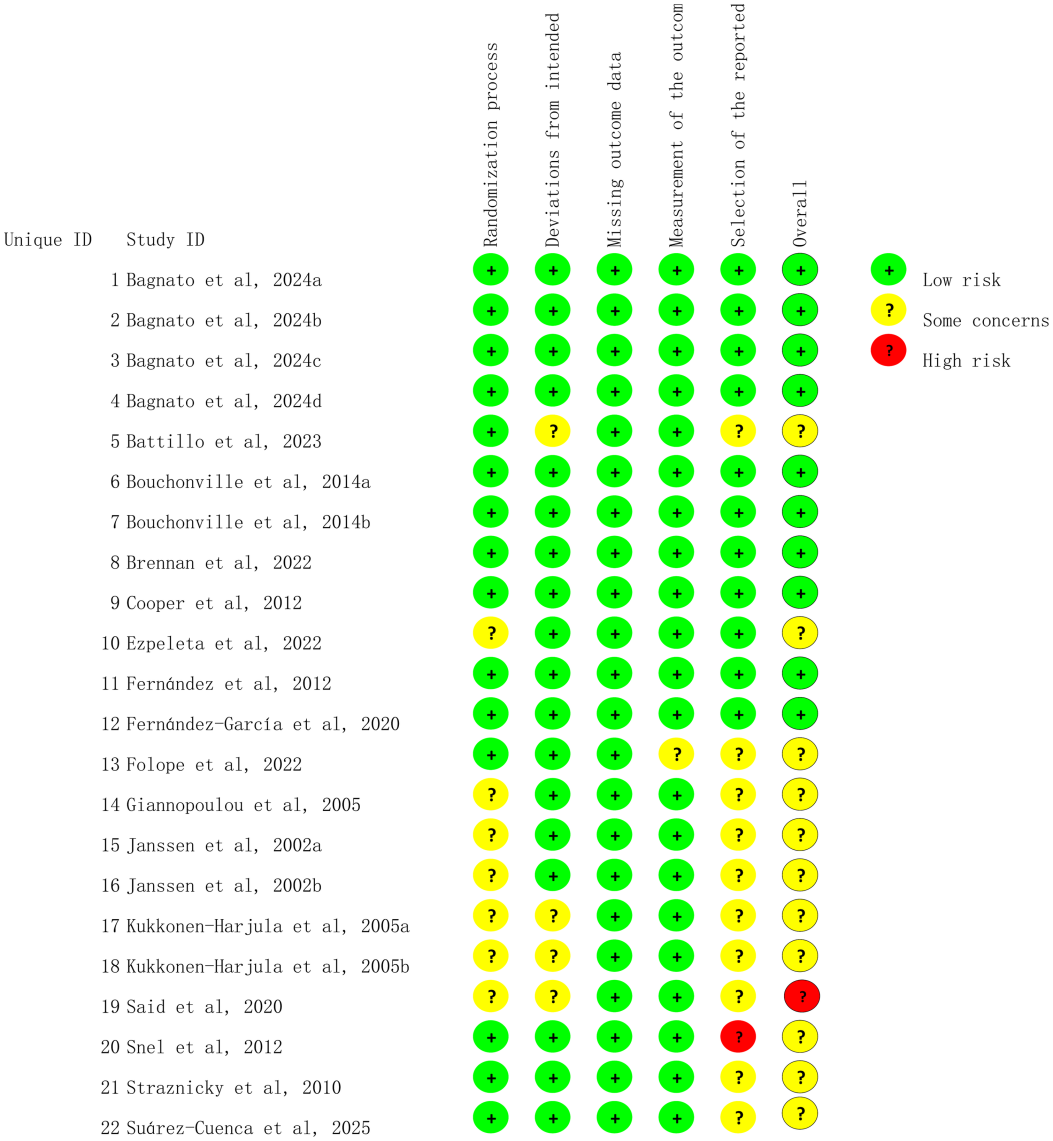
Effect of DI vs. DI + E intervention on TG.

The within-group analysis found that DI [SMD = −0.46, 95% CI: (−0.85, −0.07), *p* < 0.001, *I*^2^ = 85%] and DI + Ex [SMD = −0.77, 95% CI: (−1.15, −0.39), *p* < 0.001, *I*^2^ = 82%] resulted in significant improvements in TG (Table 2). However, directly comparing DI and DI + Ex revealed no difference between groups [SMD = 0.31, 95% CI: (−0.06, 0.69), *p* = 0.10, *I*^2^ = 83%] (Figure 5).

**Figure 6:**
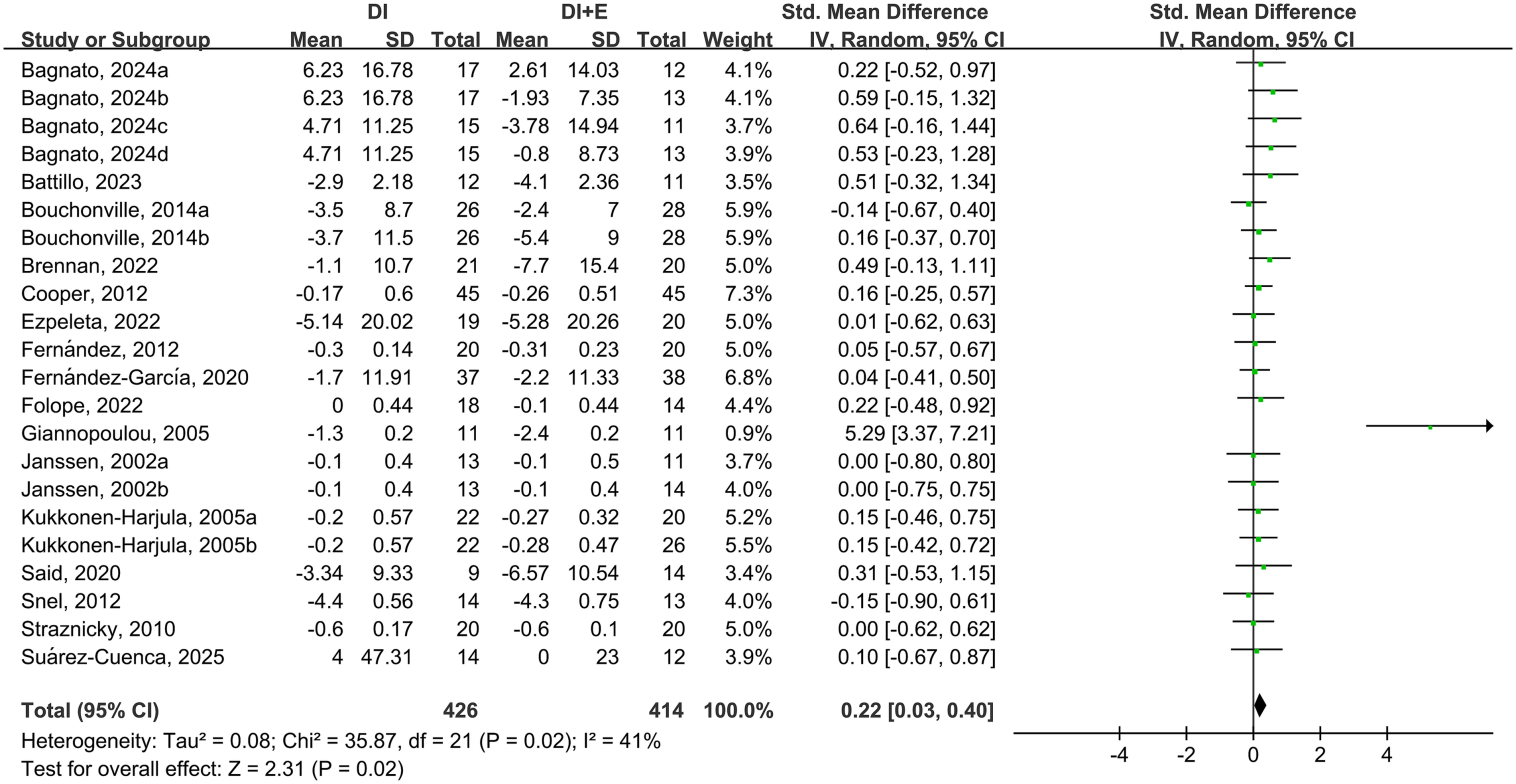
Effect of DI vs. DI + E intervention on FPG.

The within-group analysis showed that DI [SMD = −0.55, 95% CI: (−0.91, −0.19), *p* < 0.001, *I*^2^ = 84%] and DI + Ex [SMD = −0.78, 95% CI: (−1.13, −0.43), *p* < 0.001, *I*^2^ = 82%] resulted in significant improvements in FPG (Table 2). Compared with DI, DI + Ex had greater benefits for FPG [SMD = 0.22, 95% CI: (0.03, 0.40), *p* = 0.03, *I*^2^ = 41%] (Figure 6).

**Figure 7:**
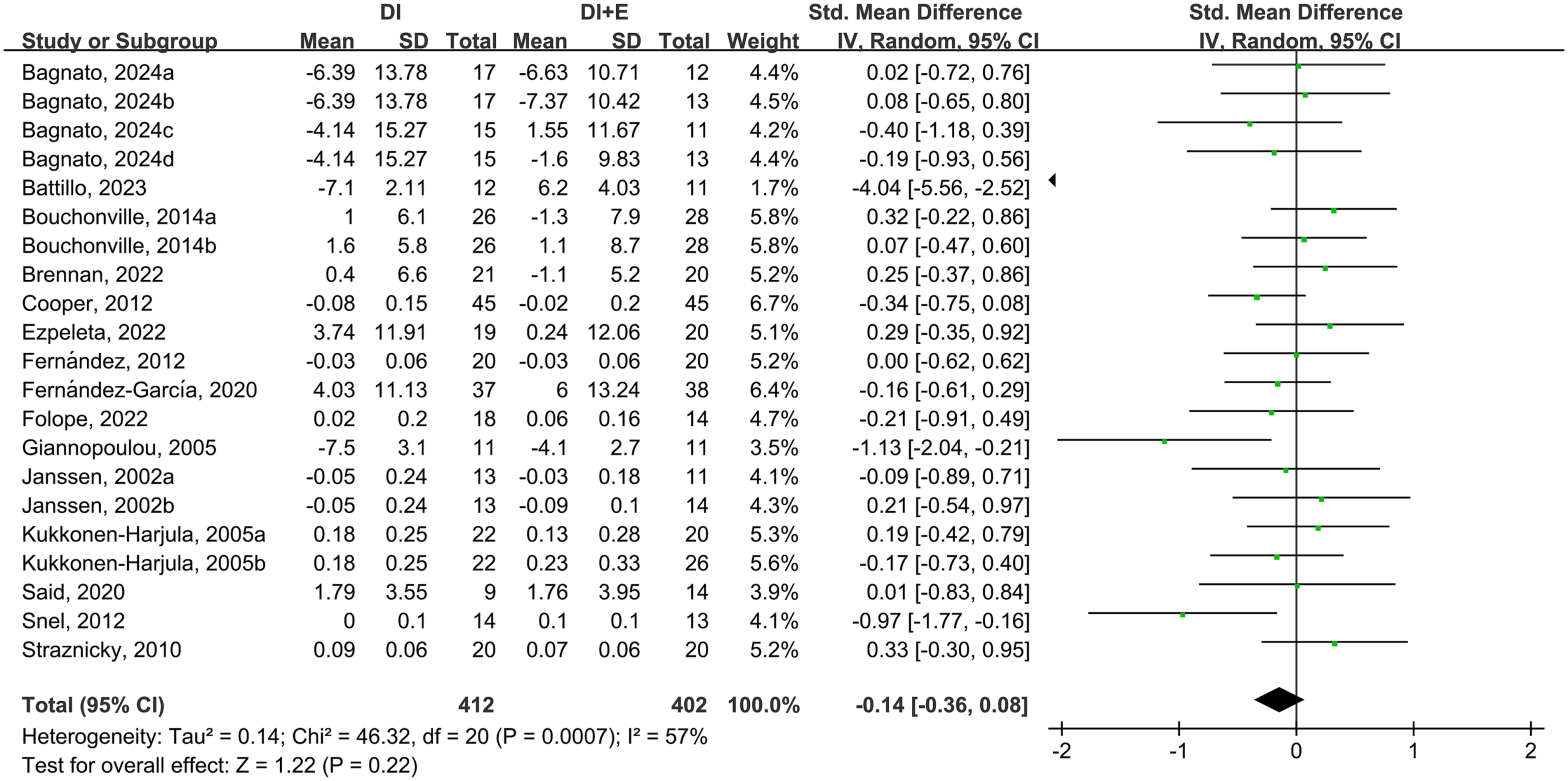
Effect of DI vs. DI + E intervention on BP.

For BP, the within-group analysis found that DI [MD = −7.92 mm Hg, 95% CI: (−12.49, −3.35), *p* < 0.001, *I*^2^ = 91%] and DI + Ex [MD = −6.81 mm Hg, 95% CI: (−11.40, −2.23), *p* < 0.001, *I*^2^ = 91%] resulted in significant improvements in SBP, whereas no difference was found between DI and DI + Ex [MD = −0.68 mm Hg, 95% CI: (−3.27, 1.91), *p* = 0.61, *I*^2^ = 66%]. The within-group analysis also showed that DI [MD = −4.50 mm Hg, 95% CI: (−6.16, −2.84), *p* < 0.001, *I*^2^ = 70%] and DI + Ex [MD = −4.84 mm Hg, 95% CI: (−7.41, −2.28), *p* < 0.001, *I*^2^ = 89%] resulted in significant improvements in DBP, and no difference was found between groups [MD = 0.33 mm Hg, 95% CI: (−2.72, 3.38), *p* = 0.83, *I*^2^ = 92%; Figure 7].

**Figure 8:**
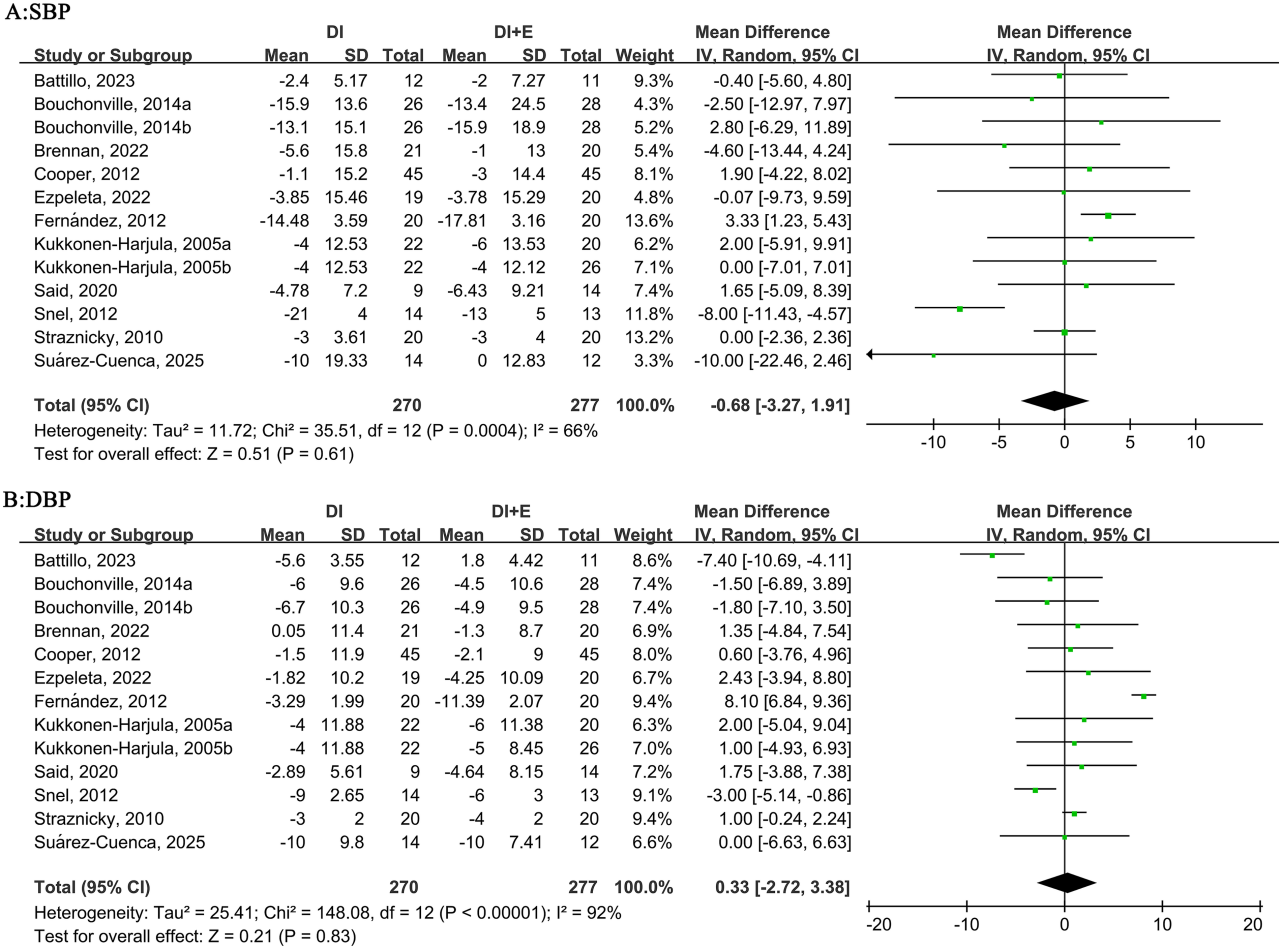
Effect of DI vs. DI + E intervention on HDL-c.

The within-group analysis revealed that DI or DI + Ex did not result in significant improvements in HDL-c and no difference between groups (Figure 8).

### Effect of DI vs. DI + ex intervention on anthropometric indices

Eighteen trials discovered significant decreases in terms of BW, 20 trials found significant reductions in BMI, and five reported changes in FFM after DI or DI + E intervention. Compared with DI, DI + Ex had greater benefits for BW [MD = 1.12 kg, 95% CI: (0.27, 1.96), *p* = 0.02, *I*^2^ = 44%], BMI [MD = 0.58 kg/m^2^, 95% CI: (0.37, 0.78), *p* < 0.001, *I*^2^ = 0%], and BF [MD = 2.27 kg, 95% CI: (0.79, 3.74), *p* < 0.001, *I*^2^ = 79%] (Table 3).

**Table 3:** Effect of DI vs. DI + Ex intervention on anthropometric index.

Outcomes	Statistical indicators		Within-group		DI vs. DI + Ex
			DI	DI + Ex	
BW (kg)	Trails		18	18	18
	MD (95% CI)		−7.00 [−9.96, −4.04]	−8.39 [−11.53, −5.24]	1.12 [0.27, 1.96]
	Heterogeneity	*I* ^2^	82	86	44
		*p*	<0.001	<0.001	0.02
	*p*		<0.001	<0.001	0.01
BMI	Trails		20	20	20
	MD (95% CI)		−2.27 [−3.35, −1.20]	−2.92 [−3.95, −1.89]	0.58 [0.37, 0.78]
	Heterogeneity	*I* ^2^	91	88	0
		*p*	<0.001	<0.001	0.82
	*p*		<0.001	<0.001	<0.001
BF (kg)	Trails		6	6	6
	MD (95% CI)		−6.68[−12.61,-0.74]	−9.06 [−17.39, −0.72]	2.27 [0.79, 3.74]
	Heterogeneity	*I* ^2^	98	95	79
		*p*	<0.001	<0.001	<0.001
	*p*		<0.001	<0.001	

BF, Body fat; BMI, Body mass index; BW, Body weight.

### Effect of DI vs. that of DI + E on blood indices

Fourteen trials discovered significant decreases in TC, 17 trials found significant decreases in LDL-c, and 13 trials reported FINs after DI or DI + Ex. The results showed that HbA1c significantly improved after DI + Ex [MD = −0.35, 95% CI: (−0.63, −0.07), *p* < 0.001, *I*^2^ = 90%] but not after DI. Compared with DI, DI + Ex had greater benefits for TC [SMD = 0.18, 95% CI: (0.00, 0.36), *p* = 0.05, *I*^2^ = 23%] and LDL-c [SMD = 0.17, 95% CI: (0.00, 0.33), *p* = 0.05] but not for FINs or HbA1c (Table 4).

**Table 4:** Effect of DI vs. DI + Ex intervention on blood index.

Outcomes	Statistical indicators		Within-group		DI vs. DI + Ex
			DI	DI + Ex	
TC	Trails		14	14	14
	SMD (95% CI)		−0.49 [−0.82, −0.16]	−0.65 [−0.96, −0.33]	0.18 [−0.00, 0.36]
	Heterogeneity	*I* ^2^	68	62	23
		*p*	0.003	0.001	0.21
	*p*		<0.001	<0.001	0.05
LDL-c	Trails		17	17	17
	SMD (95% CI)		−0.44 [−0.73, −0.14]	−0.60 [−0.86, −0.34]	0.17 [0.00, 0.33]
	Heterogeneity	*I* ^2^	68	57	11
		*p*	<0.001	0.002	0.32
	*p*		0.003	<0.001	0.05
FINs	Trails		13	13	13
	SMD (95% CI)		−1.28 [−1.77, −0.78]	−1.43 [−2.03, −0.82]	−1.28 [−1.77, −0.78]
	Heterogeneity	*I* ^2^	84	90	88
		*p*	<0.001	<0.001	<0.001
	*p*		0.01	<0.001	0.23
HbA1c	Trails		9	9	9
	SMD (95% CI)		−0.14 [−0.43, 0.15]	−0.35 [−0.63, −0.07]	−0.14 [−0.43, 0.15]
	Heterogeneity	*I* ^2^	91	88	88
		*p*	<0.001	<0.001	<0.001
	*p*		0.34	<0.001	0.08

FINs, fasting insulin; HbA1c, glycated hemoglobin; LDL-c, low-density lipoprotein cholesterol; TC, total cholesterol; TG, triglycerides.

### Subgroup analysis

Intervention duration (≤16 and >16 weeks), exercise type (AT, RT, HIIT, and CT), and participants’ age (≤ 50 and > 50 years) were subjected to subgroup analysis because of high heterogeneity. For MetS risk factors, the results showed that compared with DI, DI + Ex resulted in significant improvements in WC over long (*p* = 0.023) or short (*p* = 0.003) durations, under AT (*p* = 0.002), and in older (*p* = 0.003) or younger (*p* = 0.033) people. Subgroup analysis also revealed that compared with DI, DI + Ex resulted in significant improvements in FPG over short durations (*p* = 0.011), under AT (*p* = 0.038), under HIIT (*p* = 0.024), and in older people (*p* = 0.020). However, subgroup analysis did not reveal significant effects on TG, HDL-c, SBP, and DBP.

The results for anthropometric indices showed that compared with DI, DI + Ex resulted in significant improvements in BW over a short duration (*p* = 0.013), under AT (*p* = 0.001), and in older people (*p* = 0.023). Subgroup analysis demonstrated that compared with DI, DI + Ex resulted in a significant improvement over long (*p* = 0.005) or short (*p* < 0.001) durations, under AT (*p* < 0.001), and in older people (*p* < 0.001). Subgroup analysis showed that compared with DI, DI + Ex resulted in a significant improvement in BF over a long duration (*p* < 0.001), under AT (*p* = 0.010), under CT (*p* = 0.049), and in older people (*p* = 0.002).

For blood indices, subgroup analyses revealed a significant decrease in TC in older people (*p* = 0.018). It also revealed a significant decrease in LDL-c over a long duration (*p* = 0.018), under AT (*p* = 0.028), and in older people (*p* = 0.029). However, subgroup analysis did not reveal a significant effect on FINs and HbA1c (Supplementary File).

### Sensitivity analysis

We used sensitivity analysis to exclude the included studies individually from the overall study to assess the effect of each study on the outcome effect size and to explore the stability of results. Sensitivity analysis demonstrated that by omitting the study by Giannopoulou et al. (28), DI + Ex can significantly improve TG compared with DI. Excluding a particular study on the did not significantly affect the effect sizes of other outcomes, and the results remained stable (Supplementary File).

### Quality of studies

We examined the quality of the included studies from several aspects (Table 5).

**Table 5:** Summary of the rating quality of evidence obtained using the GRADE approach.

Outcomes	Quality assessment						Quality
	Studies (size)	Risk of bias	Inconsistency	Indirectness	Imprecision	Other considerations	
WC	630(17 studies)	Randomized trials	No serious risk of bias	No serious inconsistency	No serious indirectness	No serious imprecision	⊕ ⊕ ⊕⊕ High
TG	727(19 studies)	Randomized trials	Serious	No serious inconsistency	No serious indirectness	No serious imprecision	⊕ ⊕ ⊕⊝Moderate
HDL-c	814(21 studies)	Randomized trials	No serious risk of bias	Serious	No serious indirectness	No serious imprecision	⊕ ⊕ ⊕⊝Moderate
SBP	547(13 studies)	Randomized trials	No serious risk of bias	no serious inconsistency	No serious indirectness	No serious imprecision	⊕ ⊕ ⊕⊕ High
DBP	547(13 studies)	Randomized trials	Serious	no serious inconsistency	No serious indirectness	No serious imprecision	⊕ ⊕ ⊕⊝Moderate
FPG	840(22 studies)	Randomized trials	No serious risk of bias	No serious inconsistency	No serious indirectness	No serious imprecision	⊕ ⊕ ⊕⊕ High
BW	649(18 studies)	Randomized trials	No serious risk of bias	No serious inconsistency	No serious indirectness	No serious imprecision	⊕ ⊕ ⊕⊕ High
BMI	732(20 studies)	Randomized trials	No serious risk of bias	No serious inconsistency	No serious indirectness	No serious imprecision	⊕ ⊕ ⊕⊕ High
BFM	269(6 studies)	Randomized trials	No serious risk of bias	No serious inconsistency	No serious indirectness	No serious imprecision	⊕ ⊕ ⊕⊕ High
LDL-c	591(17 studies)	Randomized trials	No serious risk of bias	No serious inconsistency	No serious indirectness	No serious imprecision	⊕ ⊕ ⊕⊕ High
TC	478(14 studies)	Randomized trials	No serious risk of bias	No serious inconsistency	No serious indirectness	No serious imprecision	⊕ ⊕ ⊕⊕ High
FINs	495(13 studies)	Randomized trials	Serious	No serious inconsistency	No serious indirectness	No serious imprecision	⊕ ⊕ ⊕⊝Moderate
HbA1c	317(9 studies)	Randomized trials	Serious	No serious inconsistency	No serious indirectness	No serious imprecision	⊕ ⊕ ⊕⊝Moderate

BF, body fat; BMI, Body mass index; BW, Body weight; DBP, Diastolic blood pressure; FINs, Fasting insulin; FPG, Fasting plasma glucose; HbA1c, Glycated hemoglobin; HDL-c, High-density lipoprotein cholesterol; LDL-c, Low-density lipoprotein cholesterol; SBP, Systolic blood pressure; TC, Total cholesterol; TG, Triglycerides; WC, Waist circumference.

## Discussion

Meta-analysis results indicated that compared with DI alone, DI + Ex more effectively improved key MetS risk factors, WC, and FPG in individuals with obesity and MetS [SMD = 0.22, 95% CI: (0.03, 0.40), *p* = 0.03]. However, no significant effects were observed for TG, HDL-c, SBP, or DBP (*p* > 0.05). Regarding other health parameters, DI + Ex significantly improved BW [MD = 1.12 kg, 95% CI: (0.27, 1.96), *p* = 0.02], BMI [MD = 0.58 kg/m^2^, 95% CI: (0.37, 0.78), *p* < 0.001], BF [MD = 2.27 kg, 95% CI: (0.79, 3.74), *p* < 0.001], TC [SMD = 0.18, 95% CI: (0.00, 0.36), *p* = 0.05], and LDL-c [SMD = 0.17, 95% CI: (0.00, 0.33), *p* = 0.05]. By contrast, no significant improvements were found for FINs or glycated hemoglobin (HbA1c). These findings suggest that DI + Ex may offer potential advantages for improving health outcomes in individuals with obesity and MetS.

Previous reviews or meta-analyses have confirmed that Ex or DI can effectively improve health outcomes in obese individuals with insulin resistance (43–46). This finding is consistent with the statistical results of the single intervention modalities included in this study. Between-group comparative results demonstrated that compared with DI alone, DI + Ex significantly improved BW [MD = 1.12 kg, 95% CI: (0.27, 1.96), *p* = 0.02], BMI [MD = 0.58 kg/m^2^, 95% CI: (0.37, 0.78), *p* < 0.001], BFM [MD = 2.27 kg, 95% CI: (0.79, 3.74), *p* < 0.001], and WC [MD = 2.11 cm, 95% CI: (0.99, 3.23), *p* = 0.03]. These findings align with the results of previous research (47). A primary reason for this consistency is that adding Ex to DI further increases energy expenditure. Additionally, DI alone may lead to a reduction in skeletal muscle mass, which can further decrease basal metabolic rate (48). By contrast, the combination of DI + Ex effectively prevents the loss of skeletal muscle (12, 49). Furthermore, subgroup analysis revealed that short-term (≤16 weeks) interventions produced more significant effects compared with long-term (>16 weeks) interventions. These effects may be related to physiological adaptations that occur with prolonged exercise. Chen et al. found that a 2-week combined DI + Ex intervention effectively improved health parameters in individuals with MetS (50). Roberts et al. reported that a 3-week combined intervention reduced the prevalence of MetS by 60% (51). Additionally, subgroup analysis indicated that AT+DI significantly improved body composition indicators, whereas other forms of exercise did not yield significant effects. This finding is consistent with the results of Wewege et al. (52).

In terms of cardiac health markers, such as BP and blood lipids, DI and combined DI + Ex effectively improved SBP, DBP, TG, TC, and LDL-c; this finding is consistent with the results of previous meta-analyses (14, 53, 54). However, further between-group comparisons revealed that, apart from TC and LDL-c, other indicators showed no significant differences, a finding also reported by Khalafi et al. (55). A possible explanation for this discrepancy is that any intervention modality may have a ceiling effect on improvement. Not all indicators in each included study were abnormal because some fell within the normal range. Therefore, interventions produced more significant improvements in abnormal indicators than in normal ones, suggesting that the baseline values of the indices are closely related to intervention outcomes (55, 56). The analysis of anthropometric indices further supports the hypothesis that when all relevant indicators were within abnormal ranges, the DI + Ex intervention showed more significant effects than diet alone, as evidenced by metrics such as BW and BMI. The specific mechanisms behind this effect remain to be further investigated. Additionally, owing to the high heterogeneity in certain outcome indicators, we conducted subgroup and sensitivity analyses to identify potential sources. Although the exact origins of heterogeneity remain unclear, the results appear to depend partly on participant characteristics, such as age. This finding suggests that age plays a vital role in lipid profile changes. In addition to common exercise interventions, lifestyle interventions may modulate lipid metabolism, which is tightly linked to the cardiometabolic continuum. Researchers found that intensive lipid-lowering with small interfering RNA (siRNA) therapy leads not only to substantial LDL-c reduction but also to improved vascular function and arterial stiffness (57).

In terms of glycemic metabolism indicators, DI and DI + Ex effectively improved FPG and FINs. Between-group comparisons showed that compared with DI alone, DI + Ex led to greater improvement in FPG but not to a statistically significant improvement in FINs, a finding that is consistent with previous results (16, 58). The favorable effect of DI + Ex on glucose metabolism may be attributed to increased energy expenditure and reduced BF resulting from DI + Ex (59). We also found that DI + Ex was more effective than DI alone in improving BF (MD = 2.27 kg, *p* < 0.001). Abnormal lipid metabolism often leads to lipid accumulation in skeletal muscle, which can further impair insulin signaling and adversely affect glucose metabolism (60). While DI alone effectively reduces BW, it does not significantly enhance mitochondrial oxidative capacity in skeletal muscle (61). By contrast, Ex has been shown to improve mitochondrial oxidative function (62). Subgroup analysis further revealed that DI + AT and DI + HIIT were more effective than DI alone in improving FPG. A possible explanation is that AT and HIIT are more effective than other exercise modalities in reducing visceral adipose tissue (62, 63), whereas RT does not significantly improve visceral fat levels (64). In addition, studies also found that alterations in TG metabolism and HDL-c function represent early determinants of vascular injury in individuals with metabolic disturbances (65), DI + Ex can effectively improve TG, which suggests that DI + Ex can also effectively improve vascular health.

## Limitations

Our study has several limitations. First, its results showed heterogeneity, which may be introduced by differences in exercise program (type, duration, intensity, and frequency), DI characteristics, and population cohorts. Second, aerobics was the most prevalent exercise; other exercise types are needed in future studies. Third, DI protocols exhibited heterogeneity in terms of energy restriction intensity and dietary composition. Fourth, the geographic scope of the included studies is narrow, as the population is largely restricted to developed countries and lacks representation from key regions such as East Asia and Africa. First, the age range of participants (mean age from 44 to over 70 years) is high, which may limit the applicability of the results to younger adult populations. Finally, SMDs were calculated for inconsistent data units in some outcomes. However, their actual clinical importance is unknown.

## Conclusion

Our findings demonstrate that DI + Ex interventions yield significant improvements in multiple MetS risk factors among individuals with obesity. We recommend that healthcare providers and public health initiatives prioritize these integrated programs to optimize cardiometabolic health outcomes in this population.

## Data Availability

The original contributions presented in the study are included in the article/Supplementary material, further inquiries can be directed to the corresponding author.
