# Supplementary material for: Comparison of the impact of exercise training combined with dietary intervention vs. dietary intervention alone in patients with obesity and metabolic syndrome—a systematic review
Source: Front Nutr. 2025 Dec 8;12:1703600. doi: 10.3389/fnut.2025.1703600 (PMC12719256; doi:10.3389/fnut.2025.1703600)
Supplement: Supplementary file 1 [file Table_1.DOCX]

Table 1 Sub-analysis of the outcome

| **Outcomes** | **Moderators** | **Trials** | **SMD/MD**  **(95% CI)** | ***P-value*** | **Heterogeneity** | |
| --- | --- | --- | --- | --- | --- | --- |
|  |  |  |  |  | ***I^2^* (%)** | ***P*** |
| WC (cm) |  |  |  |  |  |  |
| Duration (weeks) | ＞16 | 5 | 1.82 [0.25, 3.86] | 0.023 | 0 | 0.660 |
|  | ≤16 | 12 | 2.37 [0.83, 3091] | 0.003 | 57 | 0.007 |
| Exercise Type | AT | 9 | 2.50 [0.90, 4.09] | 0.002 | 66 | 0.002 |
|  | RT | 1 | 1.00 [-1.71, 3.71] | 0.480 | - | - |
|  | HIIT | 3 | 3.05 [-1.29, 7.40] | 0.170 | 0 | 0.670 |
|  | CT | 4 | 0.94 [-1.34, 3.21] | 0.420 | 0 | 0.710 |
| Age (years) | ＞50 | 11 | 2.62 [0.89, 4.35] | 0.003 | 58 | 0.006 |
|  | ≤50 | 6 | 1.43[0.12, 2.75] | 0.033 | 0 | 0.758 |
| HDL-c |  |  |  |  |  |  |
| Duration (weeks) | ＞16 | 8 | -0.04[-0.23, 0.15] | 0.669 | 0 | 0.535 |
|  | ≤16 | 13 | -0.35[-0.83, 0.13] | 0.157 | 82 | <0.001 |
| Exercise Type | AT | 12 | -0.12[-0.34, 0.10] | 0.276 | 31 | 0.087 |
|  | RT | 2 | -0.03[-0.47, 0.42] | 0.907 | 0 | 0.420 |
|  | HIIT | 3 | -1.32[-3.86, 1.22] | 0.309 | 96 | <0.001 |
|  | CT | 4 | 0.09[-0.22, 0.39] | 0.578 | 0 | 0.677 |
| Age (years) | ＞50 | 12 | -0.08[-0.29, 0.14] | 0.486 | 26 | 0.106 |
|  | ≤50 | 9 | -0.35[-1.05, 034] | 0.319 | 90 | <0.001 |
| TG |  |  |  |  |  |  |
| Duration (weeks) | ＞16 | 6 | 0.22[-0.46, 0.90] | 0.527 | 90 | <0.001 |
|  | ≤16 | 13 | 0.36[-0.14, 0.86] | 0.158 | 83 | <0.001 |
| Exercise Type | AT | 11 | 0.56[-0.07, 1.12] | 0.079 | 90 | <0.001 |
|  | RT | 1 | -0.14[-0.87, 0.60] | 0.719 | - | - |
|  | HIIT | 3 | 0.07[-0.39, 0.52] | 0.776 | 15 | 0.303 |
|  | CT | 4 | -0.08[-0.43, 0.27] | 0.651 | 20 | 0.252 |
| Age (years) | ＞50 | 13 | 0.33[-0.16, 0.82] | 0.189 | 86 | <0.001 |
|  | ≤50 | 6 | 0.28[-0.43, 0.98] | 0.442 | 85 | <0.001 |
| SBP (mmHg) |  |  |  |  |  |  |
| Duration (weeks) | ＞16 | 6 | 0.37[-2.84, 3.59] | 0.820 | 0 | 0.826 |
|  | ≤16 | 7 | -1.24[-4.86, 2.38] | 0.502 | 80 | <0.001 |
| Exercise Type | AT | 7 | -0.75[-4.32, 2.82] | 0.681 | 79 | <0.001 |
|  | RT | 1 | -0.00[-3.99, 6.99] | 1.00 | - | - |
|  | HIIT | 2 | -3.31[-11.97, 6.99] | 0.453 | 46 | 0.174 |
|  | CT | 3 | 1.08[-3.89, 6.06] | 0.668 | 0 | 0.744 |
| Age (years) | ＞50 | 7 | -1.98[-6.04, 2.07] | 0.338 | 81 | <0.001 |
|  | ≤50 | 6 | 0.76[-2.00, 3.51] | 0.591 | 0 | 0.990 |
| DBP (mmHg) |  |  |  |  |  |  |
| Duration (weeks) | ＞16 | 6 | 0.11[-2.14, 2.35] | 0.927 | 0 | 0.923 |
|  | ≤16 | 7 | 0.41[-3.54, 4.35] | 0.840 | 95 | <0.001 |
| Exercise Type | AT | 7 | 1.85[-1.27, 4.97] | 0.245 | 90 | <0.001 |
|  | RT | 1 | 1.00[-4.76, 6.77] | 0.734 | - | - |
|  | HIIT | 2 | -4.32[-11.47, 2.83] | 0.236 | 73 | 0.054 |
|  | CT | 3 | -0.71[-3.92, 2.50] | 0.665 | 0 | 0.647 |
| Age (years) | ＞50 | 7 | 0.87[-2.41, 4.15] | 0.602 | 91 | <0.001 |
|  | ≤50 | 6 | -0.47[-4.01, 3.15] | 0.801 | 65 | 0.004 |
| FPG |  |  |  |  |  |  |
| Duration (weeks) | ＞16 | 8 | 0.14[-0.05, 0.32] | 0.151 | 0 | 0.920 |
|  | ≤16 | 14 | 0.26[0.06, 0.45] | 0.011 | 0 | 0.900 |
| Exercise Type | AT | 12 | 0.19[0.01, 0.36] | 0.038 | 0 | 0.890 |
|  | RT | 2 | 0.10[-0.35, 0.54] | 0.672 | 0 | 0.747 |
|  | HIIT | 4 | 0.43[0.06, 0.81] | 0.024 | 0 | 0.795 |
|  | CT | 4 | 0.10[-0.21, 0.40] | 0.539 | 0 | 0.750 |
| Age (years) | ＞50 | 13 | 0.25[0.04, 0.47] | 0.020 | 27 | 0.852 |
|  | ≤50 | 9 | 0.15[-0.05, 0.36] | 0.144 | 0 | 0.993 |
| BW |  |  |  |  |  |  |
| Duration (weeks) | ＞16 | 5 | 0.68[-1.18, 2.54] | 0.472 | 56 | 0.057 |
|  | ≤16 | 13 | 1.33[0.28, 2.38] | 0.013 | 49 | 0.050 |
| Exercise Type | AT | 10 | 1.81[0.71, 2.91] | 0.001 | 60 | 0.006 |
|  | RT | 1 | 0[-2.61, 2.61] | 1.000 | - | - |
|  | HIIT | 3 | -0.04[-4.05, 3.98] | 0.986 | 0 | 0.891 |
|  | CT | 4 | -0.65[-2.17, 0.87] | 0.400 | 0 | 0.681 |
| Age (years) | ＞50 | 11 | 1.57[0.22, 2.92] | 0.023 | 58 | 0.038 |
|  | ≤50 | 7 | 0.25[-0.68, 1.18] | 0.600 | 9 | 0.664 |
| BMI |  |  |  |  |  |  |
| Duration (weeks) | ＞16 | 6 | 0.74[0.22, 1.25] | 0.005 | 0 | 0.601 |
|  | ≤16 | 14 | 0.55[0.32, 0.77] | <0.001 | 0 | 0.754 |
| Exercise Type | AT | 12 | 0.64[0.42, 0.86] | <0.001 | 0 | 0.598 |
|  | RT | 2 | 0.02[-0.78, 0.82] | 0.963 | 0 | 0.601 |
|  | HIIT | 4 | 0.21[-1.07, 1.49] | 0.750 | 0 | 0.835 |
|  | CT | 2 | 0.35[-0.59, 1.29] | 0.462 | 0 | 0.664 |
| Age (years) | ＞50 | 11 | 0.75[0.46, 1.03] | <0.001 | 10 | 0.764 |
|  | ≤50 | 9 | 0.19[-0.23, 0.60] | 0.375 | 0 | 0.977 |
| BFM |  |  |  |  |  |  |
| Duration (weeks) | ＞16 | 3 | 2.59[1.31, 3.88] | <0.001 | 0 | 0.648 |
|  | ≤16 | 3 | 2.12[-0.80, 5.03] | 0.154 | 93 | <0.001 |
| Exercise Type | AT | 5 | 2.32[0.55, 4.08] | 0.010 | 84 | <0.001 |
|  | RT | 0 | - | - | - | - |
|  | HIIT | 0 | - | - | - | - |
|  | CT | 1 | 2.25[0.01, 4.49] | 0.049 | - | - |
| Age (years) | ＞50 | 3 | 3.29[1.17, 5.42] | 0.002 | 72 | 0.019 |
|  | ≤50 | 3 | 1.22[-0.47, 2.91] | 0.156 | 65 | 0.044 |
| TC |  |  |  |  |  |  |
| Duration (weeks) | ＞16 | 3 | 0.27[-0.07, 0.61] | 0.117 | 15 | 0.301 |
|  | ≤16 | 11 | 0.13[-0.14, 0.40] | 0.339 | 32 | 0.128 |
| Exercise Type | AT | 9 | 0.26[-0.01, 0.54] | 0.059 | 36 | 0.098 |
|  | RT | 1 | -0.56[-1.31, 0.19] | 0.141 | - | - |
|  | HIIT | 2 | 0.09[ -0.41, 0.59] | 0.728 | 0 | 0.874 |
|  | CT | 2 | 0.15[-0.37, 0.67] | 0.575 | 0 | 0.846 |
| Age (years) | ＞50 | 8 | 0.39[0.14, 0.64] | 0.002 | 0 | 0.674 |
|  | ≤50 | 6 | -0.07[-0.38, 0.24] | 0.650 | 26 | 0.194 |
| LDL-c |  |  |  |  |  |  |
| Duration (weeks) | ＞16 | 5 | 0.30[0.05, 0.54] | 0.018 | 0 | 0.894 |
|  | ≤16 | 12 | 0.07[-0.18, 0.31] | 0.601 | 27 | 0.126 |
| Exercise Type | AT | 10 | 0.22[0.02, 0.42] | 0.028 | 0 | 0.228 |
|  | RT | 2 | 0.001[-0.72, 0.72] | 0.999 | 59 | 0.118 |
|  | HIIT | 3 | -0.13[-0.58, 0.30] | 0.541 | 5 | 0.317 |
|  | CT | 2 | 0.36[-0.17, 0.89] | 0.179 | 0 | 0.939 |
| Age (years) | ＞50 | 8 | 0.28[0.03, 0.53] | 0.029 | 0 | 0.329 |
|  | ≤50 | 9 | 0.09[-0.12, 0.29] | 0.417 | 0 | 0.260 |
| FINs |  |  |  |  |  |  |
| Duration (weeks) | ＞16 | 5 | 0.13[-0.12, 0.37] | 0.302 | 0 | 0.985 |
|  | ≤16 | 8 | 0.53[-0.72, 1.78] | 0.404 | 95 | <0.001 |
| Exercise Type | AT | 9 | 0.54[-0.54, 1.61] | 0.329 | 96 | <0.001 |
|  | RT | 2 | -0.03[-0.52, 0.45] | 0.890 | 13 | 0.283 |
|  | HIIT | 1 | 0.14[-0.65, 0.93] | 0.726 | - | - |
|  | CT | 1 | 0.17[-0.52, 0.85] | 0.632 | - | - |
| Age (years) | ＞50 | 5 | 1.07[-0.85, 2.98] | 0.275 | 96 | <0.001 |
|  | ≤50 | 8 | 0.02[-0.20, 0.23] | 0.873 | 0 | 0.658 |
| HbA1c |  |  |  |  |  |  |
| Duration (weeks) | ＞16 | 2 | 0.43[-0.07, 0.93] | 0.092 | 43 | 0.185 |
|  | ≤16 | 7 | 0.64[-0.26, 1.54] | 0.162 | 90 | <0.001 |
| Exercise Type | AT | 7 | 0.73[-0.13, 1.60] | 0.096 | 91 | <0.001 |
|  | RT | 0 | - | - | - | - |
|  | HIIT | 2 | 0.13[-0.38, 0.63] | 0.626 | 0 |  |
|  | CT | 0 | - | - | - | - |
| Age (years) | ＞50 | 8 | 0.67[-0.05, 1.39] | 0.067 | 88 | <0.001 |
|  | ≤50 | 1 | -0.10[-0.72, 0.52] | 0.749 | - | - |

BF: body fat; BMI: body mass index; BW: body weight; DBP: diastolic blood pressure; FINs: fasting insulin; FPG: fasting plsma glucose; HbA1c: glycated hemoglobinc; HDL-c: high-density lipoprotein cholesterol; LDL-c: low-density lipoprotein cholesterol; SBP: systolic blood pressure; TC: total cholesterol; TG: triglycerides; WC: waist circumference;


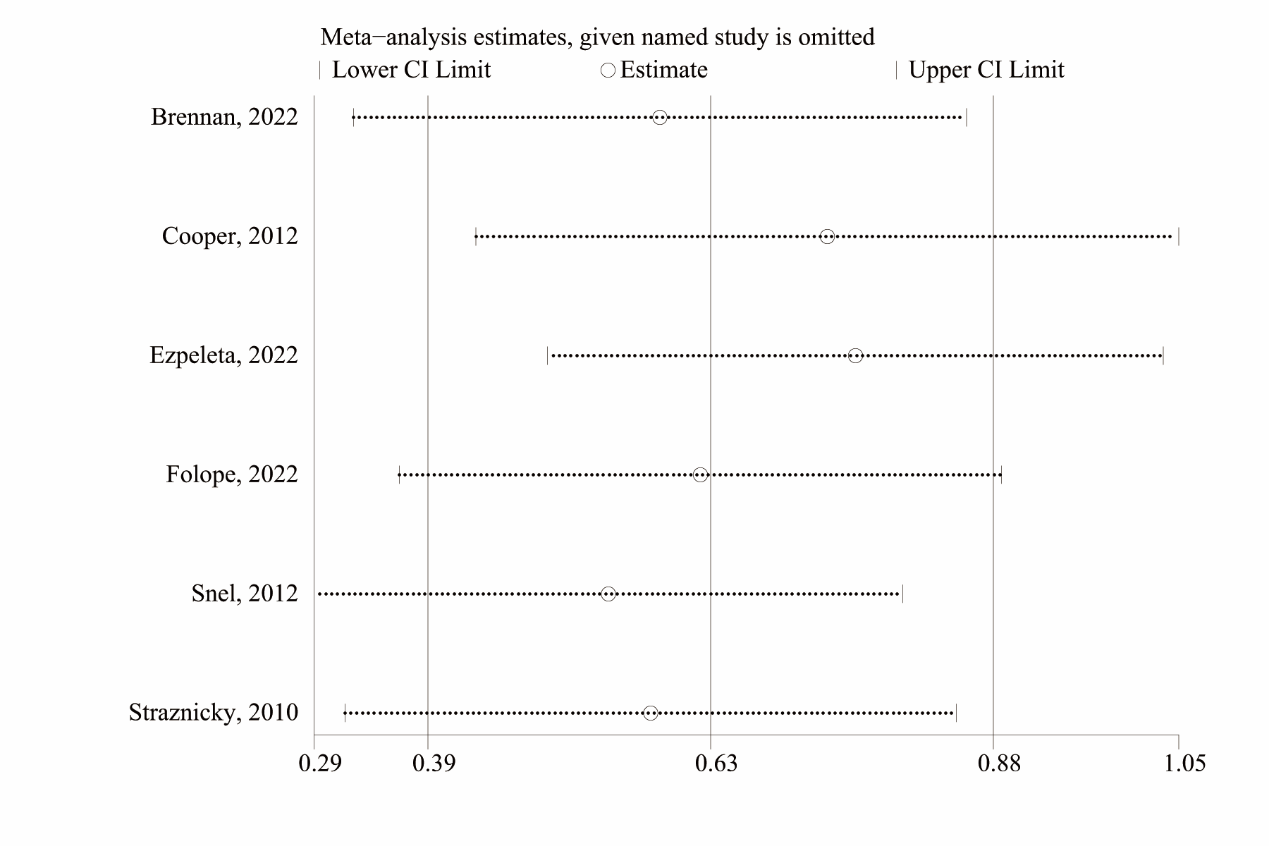


Figure 1 Sensitivity analysis of BF


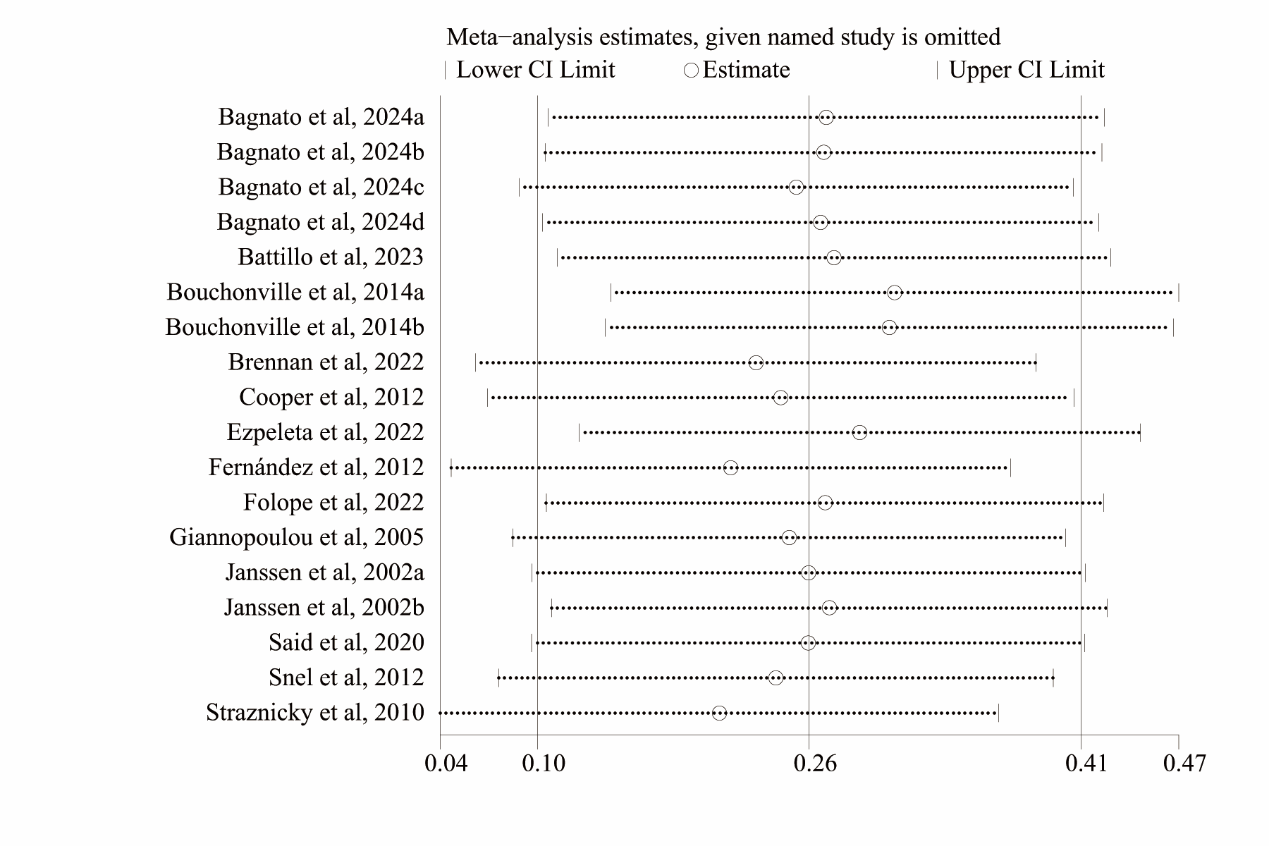


Figure 2 Sensitivity analysis of BW


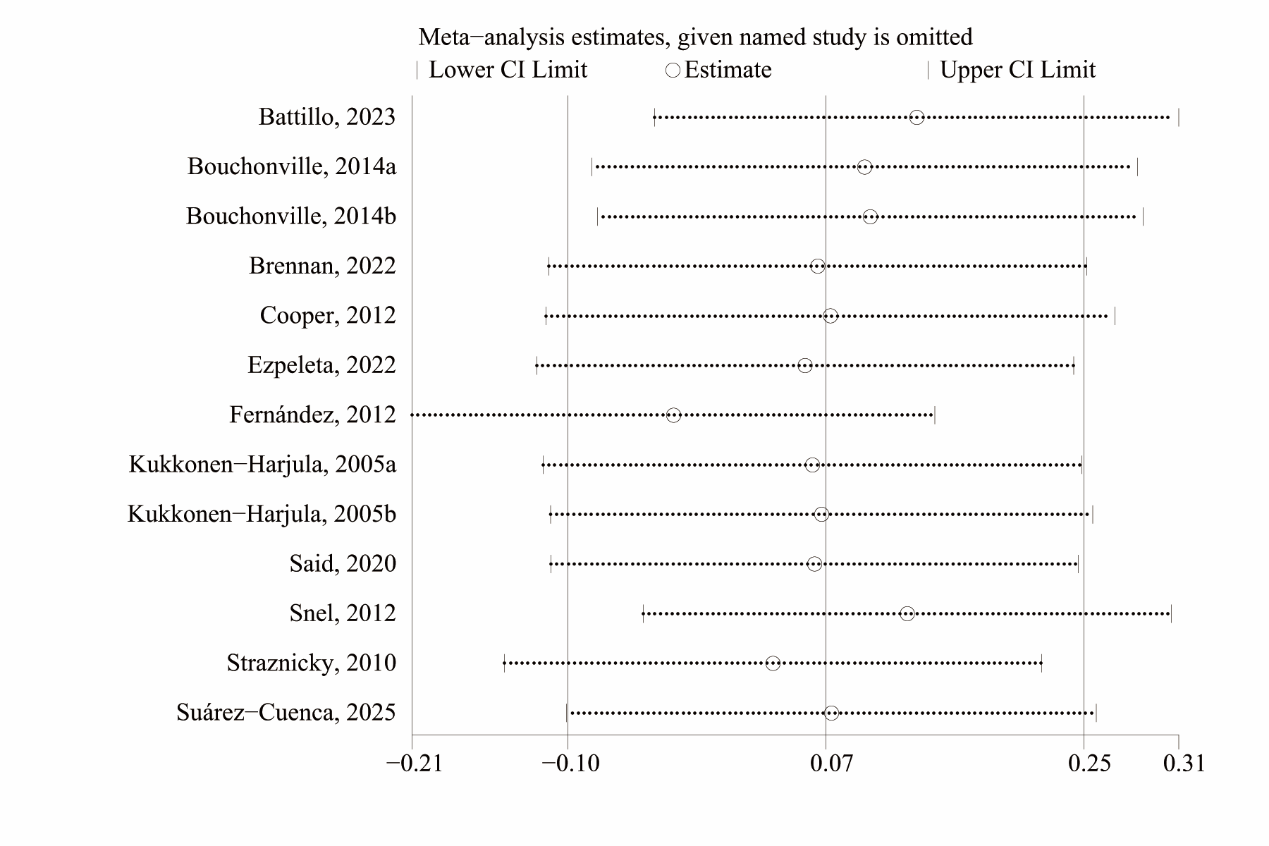


Figure 3 Sensitivity analysis of DBP


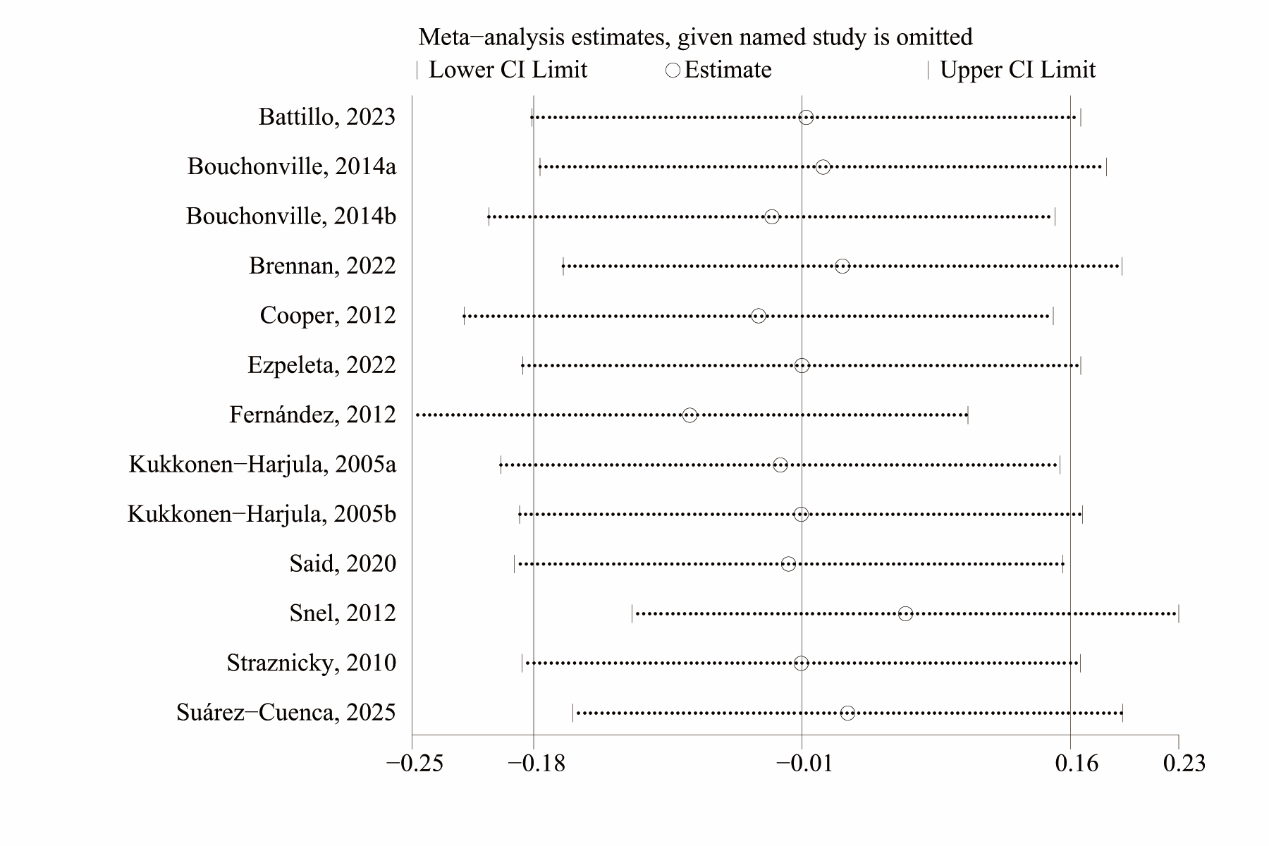


Figure 4 Sensitivity analysis of SBP


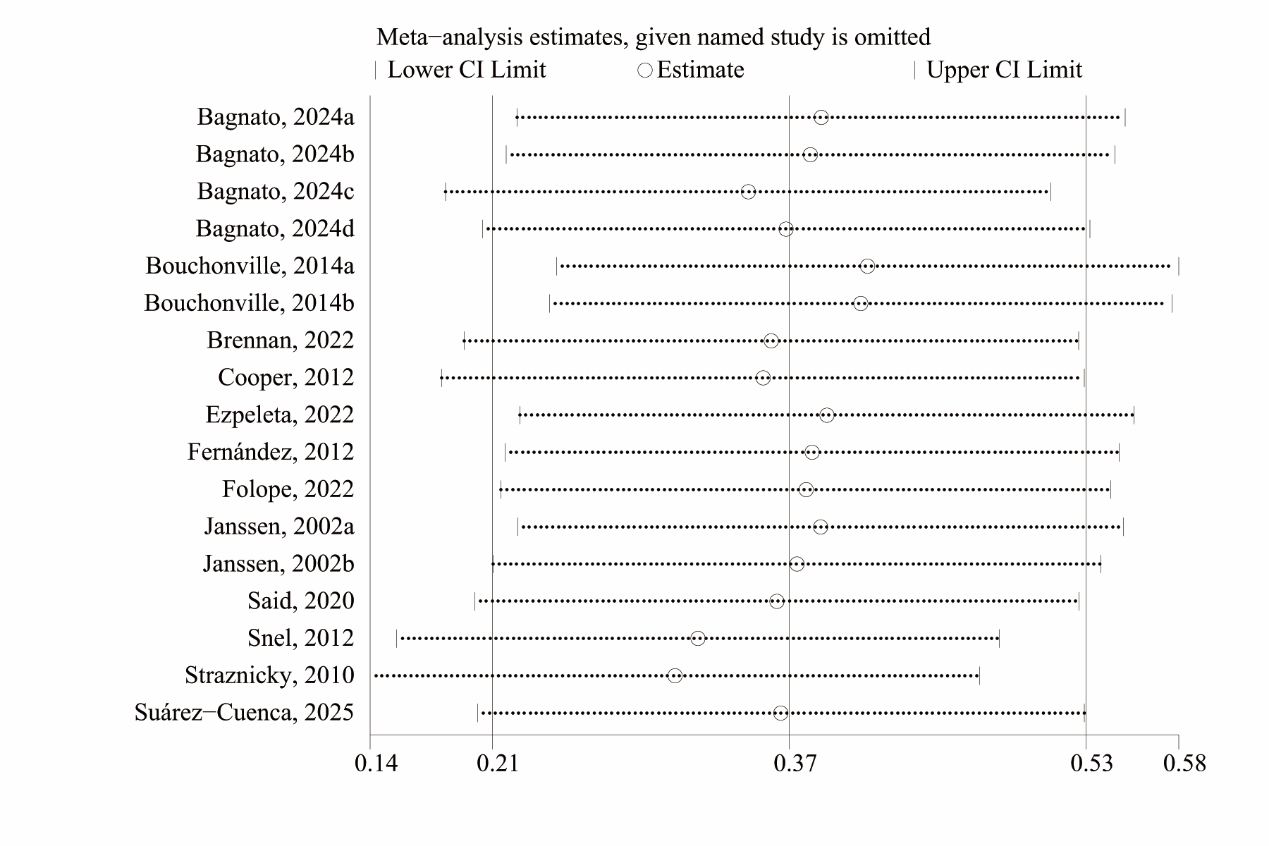


Figure 5 Sensitivity analysis of WC
